# Construction of Glucose-6-Phosphate Dehydrogenase Overexpression Strain of *Schizochytrium* sp. H016 to Improve Docosahexaenoic Acid Production

**DOI:** 10.3390/md21010017

**Published:** 2022-12-26

**Authors:** Yumei Feng, Yuanmin Zhu, Zhendong Bao, Bohan Wang, Tingting Liu, Huihui Wang, Tianyi Yu, Ying Yang, Longjiang Yu

**Affiliations:** 1 Department of Biotechnology, Institute of Resource Biology and Biotechnology, College of Life Science and Technology, Huazhong University of Science and Technology, Wuhan 430074, China; 2 Key Laboratory of Molecular Biophysics, Ministry of Education, Wuhan 430074, China; 3 Hubei Engineering Research Center for both Edible and Medicinal Resources, Wuhan 430074, China

**Keywords:** *Schizochytrium* sp., glucose-6-phosphate dehydrogenase, docosahexaenoic acid, NADPH, polyunsaturated fatty acid

## Abstract

Docosahexaenoic acid (DHA) is an important omega-3 polyunsaturated fatty acid (PUFA) that plays a critical physiological role in human health. *Schizochytrium* sp. is considered an excellent strain for DHA production, but the synthesis of DHA is limited by the availability of nicotinamide adenine dinucleotide phosphate (NADPH). In this study, the endogenous glucose-6-phosphate dehydrogenase (*G6PD*) gene was overexpressed in *Schizochytrium* sp. H016. Results demonstrated that *G6PD* overexpression increased the availability of NADPH, which ultimately altered the fatty acid profile, resulting in a 1.91-fold increase in DHA yield (8.81 g/L) and increased carbon flux by shifting it from carbohydrate and protein synthesis to lipid production. Thus, *G6PD* played a vital role in primary metabolism. In addition, *G6PD* significantly increased DHA content and lipid accumulation by 31.47% and 40.29%, respectively. The fed-batch fermentation experiment results showed that DHA production reached 17.01 g/L in the overexpressing G6PD strain. These results elucidated the beneficial effects of NADPH on the synthesis of PUFA in *Schizochytrium* sp. H016, which may be a potential target for metabolic engineering. Furthermore, this study provides a promising regulatory strategy for the large-scale production of DHA in *Schizochytrium* sp.

## 1. Introduction

Docosahexaenoic acid (DHA), an important omega-3 polyunsaturated fatty acid (PUFA), has attracted considerable interest [1,2,3] because of its beneficial effects on infant cognitive development and its ability to reduce the risk of hypertension, cardiovascular disease, neurodegenerative diseases, and certain types of cancers [4,5]. Consequently, DHA is extensively applied to functional food, pharmaceuticals, infant formula, and animal feed [6]. Interestingly, some studies have suggested that DHA dietary supplements may help reduce the risk of complications caused by coronavirus disease 2019 [7,8,9]. As a traditional source of DHA, deep-sea fishes obtain PUFA from marine microorganisms, especially marine microalgae and protists [10]. However, the overexploitation of marine resources and heavy metal pollution have severely limited the development of DHA from fish oils, and thus, alternative sources for sustainable DHA production have gained substantial interest [11,12]. *Schizochytrium* sp. is a unicellular heterotrophic marine organism that has remarkable lipid content and simple lipid composition and is suitable for large-scale fermentation [13,14]. *Schizochytrium* sp. is considered an excellent DHA producer and has been exploited as a commercial source for DHA production [15,16].

In *Schizochytrium* sp., two independent fatty acid synthesis pathways have been reported, with the specialized polyketide synthase (PKS) pathway responsible for the de novo synthesis of polyunsaturated fatty acid and the fatty acid synthase (FAS) pathway primarily synthesizing saturated fatty acid [17,18]. Lipid metabolism involves complex multi-level regulation, and understanding the mechanism of regulation of lipid metabolism by regulatory factors is essential for the precise regulation of lipid synthesis. Nicotinamide adenine dinucleotide phosphate (NADPH) is a crucial regulator of fatty acid accumulation in oleaginous microorganisms. PUFA is a highly reductive substance, and the synthesis of PUFA requires NADPH as a reducing force to reduce acetyl groups (CH_3_–CO–) to the growing acyl chains (CH_2_–CH_2_–) of fatty acid. In fatty acid synthesis, NADPH content directly determines the amounts of the fatty acid component; in microorganisms, when NADPH content is high, the percentage of unsaturated fatty acid components generally increases [19]. Therefore, to support fatty acid production, NADPH should be easily replenished by the regenerative system. Cytoplasmic malic enzyme (ME) was previously thought to be the sole provider of lipogenic NADPH [20]. However, the view that ME plays a key role in providing lipogenic NADPH has since been challenged [21,22,23]. Among non-photosynthetic organisms, the pentose phosphate pathway (PPP) is considered a primary source of reducing power for biosynthetic processes, such as lipogenesis and nitrogen assimilation [24]. In addition, NADPH is responsible for maintaining the redox potential necessary for protection against oxidative stress [25]. DHA synthesis in *Schizochytrium* sp. was promoted by the exogenous addition of regulators. The addition of 4 g/L malic acid to a culture medium of *Schizochytrium* sp. HX-308 at the stage of rapid lipid accumulation increased the enzymatic activity of ME and resulted in a 71.43% increase in DHA content [26]. Furthermore, when 200 mg of L *p*-aminobenzoic acid (*p*-ABA) were added to a *Schizochytrium limacinum* SR21 medium, *p*-ABA increased NADPH production through the enhanced pentose phosphate pathway (PPP) according to metabolomic analysis, and the final DHA yield increased by 33.28% [27]. Exogenous supplementation of 1 mM sesamol in a *Schizochytrium* sp. H016 fermentation medium promoted glucose-6-phosphate dehydrogenase (G6PD) enzyme activity and increased the supply of NADPH, which in turn increased DHA production by 90.76% [28]. These results suggest that modulating the availability of NADPH is a promising strategy for improving DHA production. However, exogenous additives increase the cost of industrial production and are not conducive to large-scale utilization.

Genetic engineering broadens the scope for improving the efficiency and industrial relevance of microalgal lipid fermentation, which has attracted the attention of many researchers. G6PD is the rate-limiting enzyme of PPP and plays an important role in supplying NADPH. G6PD catalyzes the first step of PPP and produces NADPH. The contribution of G6PD to the total production of NADPH in cells has been reported to be 60% [19]. It has been reported that co-expression of the NADP^+^-dependent *G6PD* gene and the acyl coenzyme a-binding protein gene in the yeast *Yarrowia lipolytica* resulted in a 41% increase in de novo lipid production and increased proportions of saturated fatty acid (SFA) and PUFA [29]. Notably, a significant difference in fatty acid metabolism was found between G6PD-sufficient and G6PD-deficient red blood cells (RBCs) during blood bank storage, and the levels of PUFA in G6PD-deficient RBCs were significantly lower than those in G6PD-sufficient RBCs [30]. Therefore, the overexpression of *G6PD* may be an effective way to increase PUFA level. However, in a study on the oleaginous microalga *Phaeodactylum tricornutum*, NADPH supply increased through *G6PD* overexpression resulted in a slight increase in the total SFA fraction but a 22.7% decrease in the proportion of PUFA [31]. These studies suggested that increasing the supply of NADPH is important to lipid synthesis regulation. Therefore, it is necessary to investigate the regulation of NADPH supply level on the synthesis of PUFA, especially DHA in *Schizochytrium* sp.

In this study, endogenous *G6PD* gene overexpression in *Schizochytrium* sp. increased NADPH supply during fermentation, resulting in a significant increase in lipid yield and DHA production. The fermentation characteristics of *G6PD* overexpression transformants and primary metabolite changes were examined during the fermentation process. In addition, the expression of key genes involved in fatty acid synthesis, the PPP pathway, and the glycolysis process was examined to explain the molecular mechanism of lipid metabolism regulation. Finally, the industrial application prospects of *G6PD* overexpression transformants were evaluated by fed-batch fermentation in a 5 L fermenter. This work provides several lines of evidence that the supplementation of NADPH by overexpression of G6PD is an effective strategy towards improving the accumulation DHA in *Schizochytrium* sp.

## 2. Results

### 2.1. Construction of G6PD Overexpression Strains in Schizochytrium sp. H016

**Figure 1:**
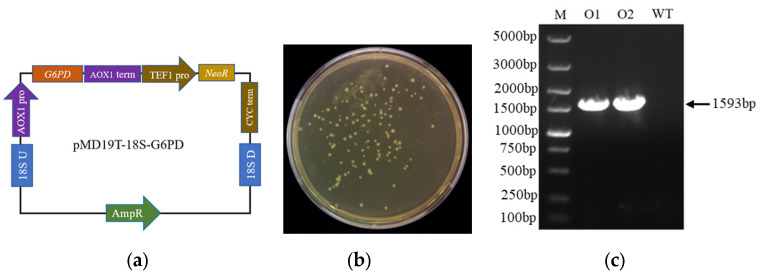
Construction of recombinant expression vectors and validation of overexpression in *Schizochytrium* sp. H016. (**a**) Vector construction scheme for the integration of *G6PD* gene expression cassette into the chromosome of *Schizochytrium* sp. H016. (**b**) Recombinant exhibited a G418-resistant phenotype, generated by the linearization of the homologous recombinant structure pMD19T-18S-G6PD targeting 18S rDNA. (**c**) Detection of recombinant integration by genomic PCR. O1-O2 and WT indicate the amplification of *neoR* fragments from the recombinants and WT, respectively.

Homologous recombination technology has been used for genome editing in eukaryotes and prokaryotes and allows the precise insertion of genes into specific locations on the host chromosome for the stable expression of target genes. In this study, a *G6PD* gene overexpression vector was constructed via homologous recombination technology, and the effects of *G6PD* overexpression on the regulation of DHA synthesis were measured. A *G6PD* overexpression vector was constructed (Figure 1a), linearized by SmaI digestion, and introduced to *Schizochytrium* sp. H016 by electroporation. G6PD-positive transformants, named OG6PD, were able to grow normally on resistant agar plates containing G418 (Figure 1b). Genomic PCR was performed on G418-resistant colonies for the amplification of the *NeoR* expression frame and the verification of the DNA insertion. A 1.59 kb band containing a *NeoR* expression cassette was amplified from the genome of the pMD19T-18S-G6PD transformant, indicating that the *G6PD* expression frame had been integrated into the chromosome of *Schizochytrium* sp. H016 (Figure 1c).

### 2.2. Validation of the Effects of G6PD Gene Overexpression in Schizochytrium sp. H016

**Figure 2:**
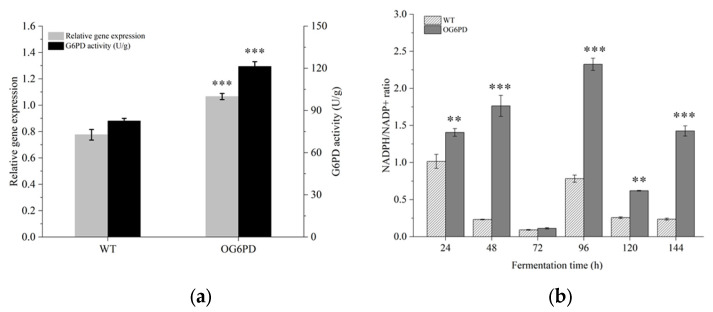
(**a**) *G6PD* transcript level and enzyme activity assay. (**b**) The changes in the intracellular NADPH/NADP^+^ ratio during fermentation. Data represent mean ± SD (*n* = 3). *p* < 0.01 and *p* < 0.001 indicate significant difference (Student’s *t*-test), which are denoted by ** and ***, respectively.

To assess the overexpression of *G6PD* gene in *Schizochytrium* sp. H016, the relative expression of *G6PD* gene and G6PD enzyme activity was measured and compared with the WT. The WT and the OG6PD were cultured in a fermentation medium and tested, and the results showed that the level of *G6PD* transcript in the engineered strain was 1.51-fold that in WT. Moreover, the activity of G6PD enzyme increased by 46.90% (Figure 2a). These results indicated that OG6PD had higher *G6PD* mRNA abundance and enzymatic activity, further confirming that *G6PD* was successfully integrated into *Schizochytrium* sp. H016.

The effect of *G6PD* overexpression on NADPH levels was investigated. The NADPH/NADP^+^ ratios of OG6PD and WT during fermentation were examined and compared. The results showed a significant difference in NADPH/NADP^+^ ratio levels between OG6PD and WT, with the NADPH/NADP^+^ ratio in the cells of OG6PD being substantially elevated during the entire fermentation process (Figure 2b). In particular, the NADPH/NADP^+^ ratio of the OG6PD increased 7.65-, 2.89-, and 5.96-fold that of WT at 48, 96, and 144 h, respectively. This result is consistent with that of a previous report, which showed that the expression of *G6PD* in *Mortierella alpina* improved NADPH level [32]. The activity of G6PD was regulated by the NADPH/NADP^+^ ratio, and when the ratio decreased, the enzyme activity of G6PD increased and promoted NADPH synthesis [33]. This conclusion was verified in the present study, where the NADPH/NADP^+^ ratio at 72 h was lowest, but the G6PD activity was enhanced by 46.9%. These results further demonstrated the effectiveness of the *G6PD* overexpression system. Consequently, the NADPH level was significantly increased in the overexpressing strains.

### 2.3. Influence of G6PD Gene Overexpression on the Fermentation Properties of Schizochytrium sp. H016

**Figure 3:**
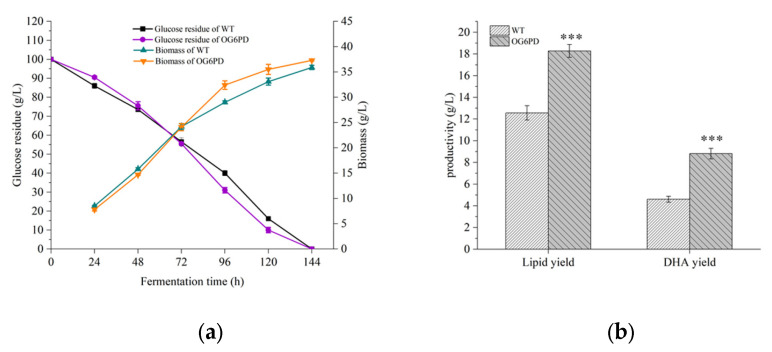
Effect of *G6PD* gene overexpression on the fermentation properties of *Schizochytrium* sp. H016. Analysis of (**a**) glucose consumption and biomass in OG6PD and WT during fermentation; (**b**) changes in lipid yield as well as DHA yield. Data represent mean ± SD (*n* = 3). Significant difference is denoted by *p* < 0.001 (***).

Oleaginous microorganism with high biomass and lipid accumulation capacities are considered important indicators for commercial production [34]. Glucose consumption and biomass were examined, and the effect of *G6PD* gene overexpression on the cellular fermentation properties of OG6PD was evaluated. OG6PD and WT showed similar trends in glucose consumption and biomass accumulation (Figure 3a). Notably, the glucose consumption rate of OG6PD was slightly accelerated, and biomass growth was correspondingly accelerated after 72 h of fermentation compared with that of WT.

The effect of *G6PD* on fatty acid synthesis was investigated in cells collected at the end of fermentation for lipid extraction, and the fatty acid profile of OG6PD and WT was analyzed by gas chromatography-mass spectrometry (GC-MS). Fatty acid composition analysis showed a significant difference between OG6PD and WT (Table 1). The proportion of total SFA decreased by 29.27% in OG6PD, and C16:0 accounted for the majority of the reduced SFA. However, the PUFA content increased by 30.09%, and DHA and docosapentaenoic acid (DPA) increased by 31.47% and 25.94%, respectively, reaching 48.17% and 11.07% of the total fatty acid content, respectively. These results clearly indicated that *G6PD* is highly associated with the fatty acid biosynthesis pathway in *Schizochytrium* sp. The overexpression of *G6PD* altered fatty acid composition and increased the percentage of PUFA, suggesting that *G6PD* is a potential target for the regulation of fatty acid composition.

Finally, at the end of fermentation, the biomass of OG6PD and WT reached 37.3 L and 35.9 g/L, respectively. In addition, the lipid and DHA yields of OG6PD reached 18.28 and 8.81 g/L, respectively, which were 45.43% and 91.11% higher than those of WT (Figure 3b). The above results suggested that the overexpression of *G6PD* effectively boosted lipid and DHA yields in *Schizochytrium* sp. H016.

### 2.4. Overexpression of G6PD Gene can Promote the Accumulation of Lipid Content

**Figure 4:**
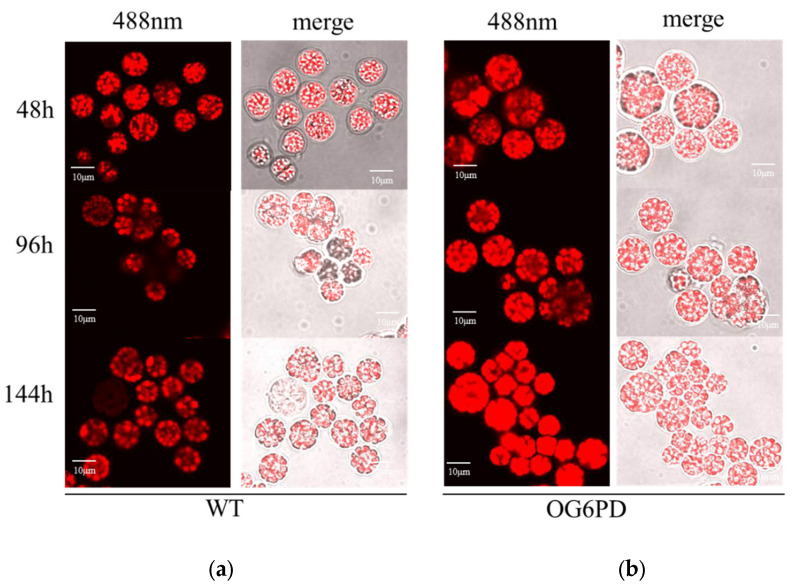
Morphological observation of *Schizochytrium* sp. H016. Cells were stained with Nile Red and then observed under laser confocal microscope. (**a**) WT, (**b**) OG6PD. Scale bar = 10 μm.

Nile Red is a dye used for rapidly detecting the abundance of neutral lipids, such as triglycerides or cholesterol esters. It binds to intracellular neutral lipid droplets and produces fluorescence that reflects the sizes of neutral lipid droplets [35,36]. To directly observe the morphology of lipid droplets and lipid distribution, OG6PD and WT were stained with Nile Red and then observed under a laser scanning confocal microscope. The findings showed that the fluorescence intensity of OG6PD was stronger than that of WT and reflected the higher neutral lipid content of OG6PD (Figure 4). The above results demonstrated that the overexpression of *G6PD* facilitated the accumulation of lipids in *Schizochytrium* sp. H016.

### 2.5. Primary Metabolite Content Was Altered with G6PD Overexpression

**Figure 5:**
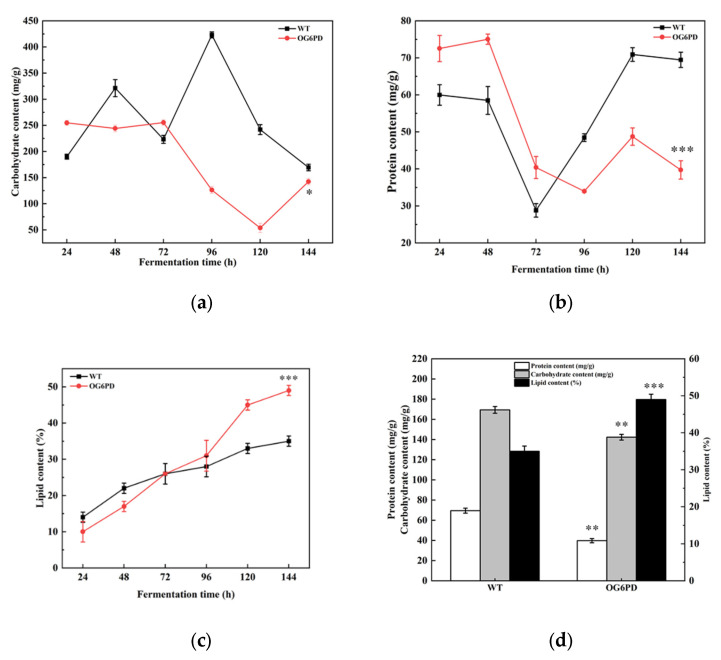
Primary metabolite product content changes during fermentation in *Schizochytrium* sp. H016. Contents of (**a**) total carbohydrate, (**b**) total protein, (**c**) total lipid. (**d**) Comparison of primary metabolite content at the end of fermentation. Data represent mean ± SD (*n* = 3). Significant differences are denoted by *p* < 0.05 (*), *p* < 0.01 (**), or *p* < 0.001 (***).

The main components of microorganism are lipids, proteins, and carbohydrates [37]. These substances are interconvertible with each other. Carbohydrates and lipids are usually the main energy storages, and changes in total protein can reflect the rate of metabolic activity in cells [38]. The impact of *G6PD* overexpression in the primary metabolism of *Schizochytrium* sp. was evaluated according to variations in total carbohydrate, lipid, and protein content during fermentation. The results revealed that the total protein and total carbohydrate content of OG6PD were 39.72 and 142.27 mg/g, respectively, which decreased by 42.82% and 16.01% at the end of fermentation (Figure 5a,b). Interestingly, the lipid content increased by 40.29% compared with that in WT (Figure 5c,d). This result was consistent with the confocal microscopy observation that OG6PD possessed a high number of lipid droplets. These results indicated that changes in lipid content due to *G6PD* in the overexpressed transformants are negatively correlated with the total carbohydrate and total protein content in the organisms. Reduction in cellular protein concentration is associated with intracellular protein degradation, which provides the nitrogen required for basic metabolic functions [39]. The above results suggested that overexpressed *G6PD* plays a critical role in primary metabolism by redirecting carbon flux from carbohydrate and protein synthesis to lipogenesis.

### 2.6. Key Enzymes’ Expression Levels Were Verified by Quantitative Real-Time PCR

**Figure 6:**
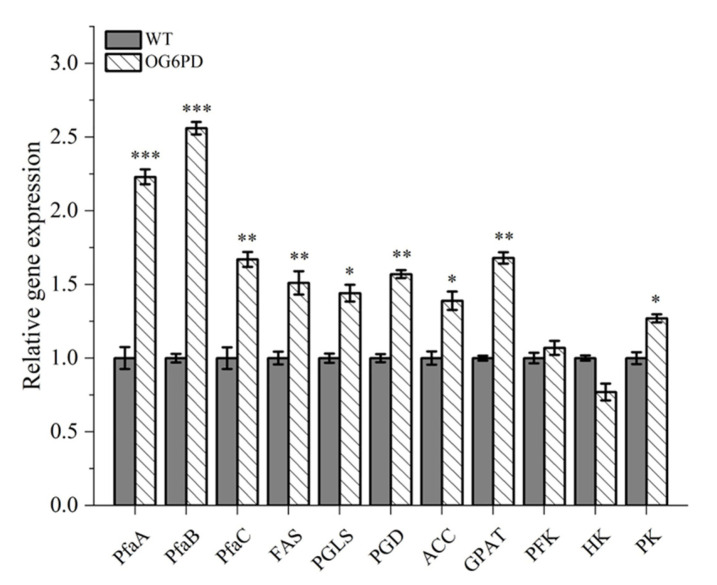
Relative expression level changes in key genes in fatty acid synthesis were analyzed by qRT-PCR at 72 h. Data represent mean ± SD (*n* = 3). Significant differences are denoted by *p* < 0.05 (*), *p* < 0.01 (**), or *p* < 0.001 (***).

The regulatory role of *G6PD* in *Schizochytrium* sp. H016 was studied by analyzing the transcriptional profiles of enzymes involved in DHA synthesis, PPP, glycolysis, and TAG synthesis in OG6PD (Figure 6). The first step in the biosynthesis of fatty acid is the formation of malonyl-CoA from acetyl-CoA catalyzed by acetyl-CoA carboxylase (ACC) [40]. Notably, the expression of the ACC gene increased 1.39-fold in the overexpression transformants compared with that in WT. The overexpression of ACC in *Schizochytrium* sp. increased the lipid and DHA yields by 30.50% and 41.86%, respectively [41].

The PPP pathway is a pathway for glucose-6-phosphate metabolism to produce NADPH [42]. 6-Phosphogluconolactonase (*PGLS*) and 6-phosphogluconate dehydrogenase (*PGD*) are the key genes in the oxidative phase of this pathway. The relative gene expression levels of *PGLS* and *PGD* increased 1.44- and 1.57-fold, respectively, in OG6PD compared with WT. The increased expression of *PGLS* and *PGD* indicates that the production of NADPH is necessary to meet energy requirements.

Four genes were used in testing the mechanism underlying the effect of *G6PD* overexpression on fatty acid biosynthesis in *Schizochytrium* sp. H016. The *FAS* gene is a multifunctional complex enzyme involved in fatty acid synthesis in the FAS pathway. *FAS* may be responsible for the synthesis of SFA, whereas *PfaA*, *PfaB*, and *PfaC*, which encode polyketide synthases, may be responsible for the synthesis of long-chain PUFA in *Schizochytrium* sp. [43]. The expression of *FAS* in OG6PD increased 1.51-fold compared with that in WT. Interestingly, *PfaA*, *PfaB*, and *PfaC* were expressed at much higher levels during fatty acid synthesis than *FAS*, and OG6PD showed gene expression levels 2.23-, 2.56-, and 1.67-fold higher than those in WT. The above results suggested that the overexpression of *G6PD* promotes the upregulation of the FAS pathway and PKS pathway genes, which in turn promotes fatty acid synthesis.

Fatty acid was assembled into TAG after several successive reactions. Glycerol-3-phosphate acyltransferase (*GPAT*) is a key gene in the biosynthesis of TAG and is responsible for the first step of TAG assembly, which is also a rate-limiting reaction [44]. Notably, the expression of *GPAT* was increased 1.68-fold in OG6PD. Similarly, *GPAT* overexpression resulted in the marked accumulation of TAG in *Cyanidioschyzon merolae* [45]. Hence, the upregulated *GPAT* gene promoted the synthesis of TAG and lipid yield.

Acetyl-CoA is mainly produced through metabolic circuits, such as glycolysis and β-oxidation and catabolism of branched-chain amino acids [46]. Therefore, an enhanced glycolytic pathway will provide more prerequisites for fatty acid synthesis. Hexokinase (*HK*), phosphofructokinase (*PFK*), and pyruvate kinase (*PK*) are involved in three irreversible reactions during glycolysis and have modulatory effects on the glycolytic pathway. The expression of *PFK* and *PK* was slightly elevated (1.07- and 1.27-fold, respectively) in OG6PD compared with WT, but the expression of *HK* decreased by 23%. The end product of glycolysis is pyruvate, which is converted to acetyl-CoA in the mitochondria. Increase in *PK* expression level may accelerate the accumulation of acetyl-CoA, which provides a large amount of reaction substrate for fatty acid production. The overexpression of *PK* promotes keratinocyte glycolytic metabolism, whereas the downregulation of *PK* inhibits keratinocyte proliferation and glycolysis [47].

The results suggested that *G6PD* manipulation is effective in promoting fatty acid synthesis and lipid production in *Schizochytrium* sp. H016, indicating that *G6PD* plays a crucial role in lipid accumulation. Therefore, *G6PD* is a potential regulatory target for regulating lipid synthesis.

### 2.7. Fed-Batch Fermentation Characterization of the OG6PD Strain

**Figure 7:**
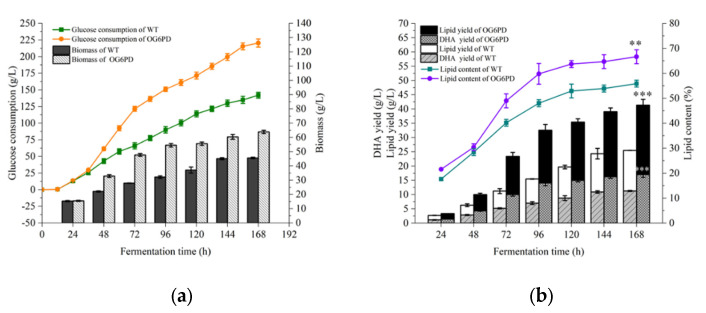
Comparison of glucose fed-batch fermentation characterization between the WT and OG6PD strains in 5 L fermenter: (**a**) glucose consumption, (**b**) DHA yield, lipid yield, and lipid content. Data represent mean ± SD (*n* = 3). The fermentation endpoint data were analyzed for significant differences by *t*-test, ** *p* < 0.01, *** *p* < 0.001.

The ability of the OG6PD strain to produce DHA-rich lipids was evaluated through pilot-scale fermentation experiments in a 5 L fermenter. As shown in Figure 7a, the OG6PD strain exhibited better growth and faster glucose consumption than the WT strain during fed-batch fermentation. At the end of fermentation (168 h), OG6PD produced the largest biomass (63.90 g/L), which was 40.13% higher than the biomass of the WT strain. Additionally, OG6PD exhibited higher lipid and DHA yields throughout the fermentation process, reaching a maximum of 41.35 and 17.01 g/L at the end of fermentation, respectively, which were 57.52% and 46.51% higher compared with the yields in WT, respectively (Figure 7b). The results indicated that the *G6PD* overexpression transformant has a good application potential for the industrial production of DHA.

## 3. Discussion

The study of key regulatory factors of microbial lipid accumulation plays an important role in alleviating the energy crisis and the unsustainable production of chemical products. NADPH is a key cofactor required for fatty acid synthesis in all organisms. Therefore, the effectiveness of NADPH may be the key to the regulation of lipid accumulation in oleaginous microorganisms. In this work, *G6PD* in the PPP pathway can regulate the supply of NADPH in *Schizochytrium* sp. H016 and promote lipid synthesis, which ultimately leads to a significant increase in DHA production (1.91-fold increase in DHA production in OG6PD compared with WT). The main reasons were remarkable improvements in DHA and lipid content.

The accumulation of lipids is a complex process influenced by the regulation of the conversion and distribution of the three main substances: carbohydrates, lipids, and proteins. The total content of protein and carbohydrate in the G6PD overexpression transformants were decreased, but the total lipid content increased (Figure 5). Fluorescence intensity in the overexpression transformants was stronger than that in the WT, as shown by confocal microscopy (Figure 4). In microalgae, carbon storage forms other than lipids include additional material forms apart from carbohydrates, and they are competing pathways because their synthesis requires common precursor substances [48,49]. Thus, enhanced lipid accumulation in the overexpressed transformants was likely a result of a metabolic shift. Glycolysis and the PPP pathway were possibly stimulated to some extent (Figure 2) and provided intermediates such as acetyl-CoA, dihydroxyacetone phosphate, and NADPH for fatty acid and lipid synthesis [50,51]. Reduced G6PD activity increases cellular sensitivity to oxidative stress, and elevated intracellular oxidants inhibit cell growth [52]. Furthermore, G6PD deficiency retards human fibroblast growth and accelerates cellular senescence [53]. In HCT116 colon cancer cells, *G6PD* knockdown reduces the NADPH/NADP^+^ ratio and oxidative stress sensitivity and inhibits growth [54]. In particular, the accumulation of TAG by cells and the response to oxidative stress require metabolic reallocations, including the delicate regulation of NADPH pool equilibria, the activation of various genes or proteins involved in lipid biosynthesis, and the interactions of other metabolisms [55]. Antioxidant systems, including the three major systems of glutathione, catalase, and superoxide dismutase, all depend on the presence of NADPH to exert their effects [56]. As a result, an increased level of NADPH supply can mitigate oxidative damage to lipids. Thus, the *G6PD* gene, as a critical gene controlling NADPH synthesis in the PPP pathway, exerts regulatory effects on both lipid accumulation and cell growth in *Schizochytrium* sp. H016.

Biomass is often the limiting factor for the industrial production of microalgae and increases in the yields of high-value-added products [57]. An interesting phenomenon was that the cell growth rate of G6PD transformants was slightly faster than that in WT during shake flask fermentation, and this result was more evident during the fermenter culture. This may be the avoidance of substrate inhibition caused by high initial glucose concentration [58]. During the fed-batch process, a substantial increase was observed in the consumption rate of glucose, the main source of carbon and energy for cell growth and survival, which provides essential intermediates, such as amino acids, fatty acids, and nucleic acids. High levels of glucose ingestion leading to the production of ATP, pyruvate, and intermediate metabolites during glycolysis. PPP is a branch of glycolysis, and its first-reaction substrate is an intermediate of the glycolytic process and produces ribose and NADPH. It is used in synthesizing precursors for nucleotide anabolism and maintaining the redox state of the cell, promoting the proliferation growth, and survival of cancer cells [59]. The upregulation of the expression levels of key enzyme genes (*PFK*, *PK*) in glycolysis explains the enhanced glucose uptake, which promotes cell growth and leads to the accumulation of higher biomass (Figure 6).

In previous studies, it was shown that the FAS pathway and the PKS pathway can produce SFAs and PUFA by using different sources of NADPH and substrates, respectively [60,61]. In this study, G6PD enzyme activity was increased by 46.90% in OG6PD (Figure 2A). The PUFA content in OG6PD increased by 30.09%, especially DHA and DPA to a more significant extent; however, the SFAs’ content decreased by 29.27% (Table 1). In addition, the gene expression of PKS synthases (*PfaA*, *PfaB*, and *PfaC*), genes related to fatty acid synthesis, was more greatly upregulated than that of FAS synthases (Figure 6). This result indicates that the NADPH produced by the PPP pathway involved in *G6PD* is closely related to the fatty acid biosynthesis process. This also supports the view that PKS synthase mainly uses NADPH produced by the PPP pathway to participate in the synthesis of PUFA.

## 4. Materials and Methods

### 4.1. Microorganisms and Culture Conditions

*Schizochytrium* sp. H016 (CCTCC M 2021260) was obtained by isolation from the area of Qingdao city, Shandong province, China. It was cultured at 28 °C and shaken at 200 rpm for 48 h on seed medium containing 50 g/L glucose, 2 g/L peptone, 5 g/L yeast extract, 1.5 g/L KH_2_PO_4_, 0.5 g/L MgSO_4_·7H_2_O, and 20 g/L seawater crystals. The seed culture was subsequently transferred to a 250 mL shake flask containing 50 mL of fermentation medium at a final concentration of 4% (*vol*/*vol*). The medium was composed of 100 g/L glucose, 3 g/L NaNO_3_, 15 g/L yeast extract, 1.5 g/L KH_2_PO_4_, 0.5 g/L MgSO_4_·7H_2_O, and 2 g/L Na_2_SO_4_. The cell was incubated at 28 °C on an orbital shaker at 200 rpm for 6 days for lipid and DHA production. The basic liquid seed medium was supplemented with 0.2 mg/mL G418 and 2% (*wt*/*vol*) agar as solid seed medium for the screening of transformants.

Fed-batch fermentation was carried out in a 5 L fermenter with a filling volume of 3 L. *Schizochytrium* sp. H016 was cultured in the seed medium for two generations and then transferred at 10% (*vol*/*vol*) into fermenters for breeding at 28 °C for 168 h. The initial glucose concentration was 50 g/L in fed-batch fermentation. In addition, glucose concentration was measured every 12 h and supplemented with 500 g/L glucose feed solution during fermentation. The stirring speed was regulated at 200–500 rpm to maintain the dissolved oxygen (DO) at 30% with an aeration rate of 1.0 *vol*/*vol*/*min* (vvm), and pH was kept at 7.0.

### 4.2. G6PD Gene Cloning, Analysis, and Plasmid Construction

A G6PD overexpression frame was integrated into the chromosome by 18S rDNA homologous recombination. The bleomycin resistance gene in pPICZαA was replaced by the neomycin resistance gene (*neoR*) in pcDNA3.1(+). The new vector, named pPICNαA-G418, was amplified by reverse PCR for the removal of the α-factor secretion signal and then digested with EcoRI/XbaI. *G6PD* was amplified from *Schizochytrium* sp. H016 (CCTCC M 2021260) cDNA and digested with EcoRI/XbaI. The digested vector and *G6PD* were ligated together with T4 DNA ligase (TaKaRa, Shiga, Japan), and the ligation reaction was completed at 16 °C and named pPICNA-G6PD. The inserted DNA fragments were examined by sequencing. The *G6PD* and *neoR* expression cassettes were amplified from this new vector, including the AOX1 promoter, *G6PD* gene, AOX1 terminator, TEF1 promoter, *neoR* gene, and CYC1 terminator. The three fragments (18S upstream, *G6PD* and *neoR* gene expression cassette, 18S downstream) were then seamlessly ligated to the pMD19-T vector with a seamless assembly cloning kit (Clone Smarter, Houston, TX, USA) according to the manufacturer’s protocol. The vector was named pMD19T-18S-G6PD. The PCR primers used in this study are provided in Table S1.

### 4.3. Validation Transformation of Schizochytrium sp. H016

With the same culture method, *Schizochytrium* sp. H016 was cultured in the seed medium until the logarithmic phase. Then, the cells were subjected to 4000 rpm centrifugation for 5 min. The newly prepared LDST solution (pH 7.5) containing 10 mmol/L Tris-HCl, 100 mmol/L LiAc, 0.6 moL/L sorbitol, 10 mmol/L DTT was added, and the strain was incubated at 28 °C for 30 min and centrifuged again under the same conditions. Washing with 1 M ice-cold sterile sorbitol was performed three times to remove the remaining medium. Finally, 1 M ice-cold-sterile-sorbitol-resuspended competent cells were used. The obtained cells were place on ice until use. Then, 3 μg of linearized vector pMD19T-18S-G6PD digested with enzyme SmaI was mixed with competent cells. The condition for the implementation of electroporation was as follows: 1400 voltage, 50 μF capacitance, 200 Ω resistance, and 2 mm cuvette. After electroporation, 1 mL of fermentation medium was immediately added to the cells, which were then cultured for 2 h in a constant temperature shaker at 28 °C and 200 rpm to revive the cells. Resuscitation cell suspension (100 μL) was spread on a solid plate of seed medium containing 0.2 mg/mL G418 antibiotics and cultured at 28 °C for 24 h. Plaques that were able to grow were selected, and their genomes were extracted for subsequent PCR verification.

### 4.4. Dry Cell Weight, Glucose Concentration, and Lipid Content Measurements

For dry cell weight detection, 50 mL of fermentation liquid were taken into a centrifuge tube and centrifuged for 10 min at the centrifugal force of 8000× *g* and 4 °C. The supernatant was removed and washed with sterile water three times. The cells were collected by centrifugation and dried in a vacuum drying oven at 80 °C for 12 h to a constant weight. The residual glucose in the fermentation broth was measured by biosensor SBA-40D. The lipid content was detected by organic solvent extraction after mechanical wall breaking. The dried cells were broken with mortar. About 0.5 g of dried cell powder was added to a 10 mL centrifuge tube, and 5 mL of petroleum ether (30–60 °C boiling range) were added. After intermittent ultrasonic treatment for 10–20 min, the supernatant was obtained by centrifugation at 8000× *g* for 10 min and then transferred to a clean centrifuge tube. The above extraction procedure was repeated three times. Finally, it was placed in vacuum drying oven at 80 °C to a constant weight.

### 4.5. Analysis of Fatty Acid Component and Determination of Neutral Properties

The fatty acid methyl esters (FAMEs) were based on Indarti et al. [62], with some modifications. Briefly, a 50 μL oil sample was taken, then 1 mL of 1 mol/L KOH–CH_3_OH (3:7) solution was added, placed at 65 °C for 30 min, and cooled to room temperature. Then, 2 mL of boron (tri) fluoride etherate were added. The solution was cooled to room temperature after reaction at 65 °C for 5 min, and 1 mL n-hexane and 1 mL saturated sodium chloride solution were added after shaking and mixing. The solution was left to stand, and the upper clear organic layer was filtered with a 0.45 μm filter membrane.

In this study, the composition of FAMEs was analyzed by GC-MS. The GC column used for the determination of FAMEs in GC-MS analysis was HP-5MS (19091S-433, 30.0 m × 250 µm) with a maximum temperature of 350 °C. The inlet temperature of the GC was set to set to 300 °C. High-purity helium was used as the carrier gas. A consistent pressure mode was used. The split ratio was 50:1. The temperature program was set as follows. the temperature was first increased at a rate starting at 50 °C with a retention time of 2 min. The temperature was increased to 150 °C at a rate of 10 °C/min, and then the temperature was increased to 270 °C at a rate of 5 °C/min. The GC-MS transfer line temperature was set to 280 °C, and GC-MS detection was performed in full scan mode. A 1 µL aliquot of each sample was injected into the GC column. A fatty acid standard mixture consisting of 37 components was used to validate the temperature procedure. The peaks of the 37 fatty acids were separated in sequence.

Nile Red is a lipophilic oxazine fluorescent dye that binds and fluoresces with lipids and facilitates the visualization of intracellular lipid droplets and neutral lipids. To detect the content of neutral lipids in *Schizochytrium* sp. H016, the protocol was modified according to the method described by Chen et al. [63]. Cell cultures were treated with PBS buffer for 20 min, and 10 μL of Nile Red dye was added to 1 mL of pretreated cells, mixed rapidly, placed in a dark environment, incubated for 20 min, and then observed by laser confocal microscopy (Olympus, Tokyo, Japan). The wavelengths used were 488 nm excitation light and 510–550 nm emission light.

### 4.6. Detection of Carbohydrate and Protein Content

Total carbohydrate acid hydrolysis was reduced to amino compounds after co-heating with DNS reagent under alkaline conditions. The products were red-brown in an alkaline solution, and the amount of reducing sugars was proportional to the shade of orange-red color of the substance, so as to determine the total carbohydrate content in the sample. In conclusion, after collection of the organisms, they were left to dry at 60 °C to a constant weight and then treated according to the total carbohydrate extraction kit’s instructions (Solarbio, Bejing, China). The protein concentrations of the samples from the same batch were measured using a whole protein extraction kit based on the principle of the bicinchoninic acid (BCA) assay method.

### 4.7. Preparation of Schizochytrium sp. H016 Cell Extracts, Determination of Enzyme Activity and NADPH Content

Fermentation broth was obtained (10 mL) and centrifuged at 8000× *g* at 4 °C for 10 min. The cell pellets were washed with ice-cold deionized water three times and then with 100 mM Tris-HCl buffer (pH 7.4) containing 20% (*w*/*v*) glycerin and 2 M DTT three times. Then, treatment with liquid nitrogen was performed to break the cell wall. The damaged cells were treated with the same buffer solution (100 mM Tris-HCl buffer) and then centrifuged at 8000× *g* at 4 °C for 10 min. The supernatant obtained was the cell extract, which was used for enzyme activity detection.

After the cell extracts were prepared, the enzyme activity of G6PD was detected according to the manufacturer’s protocols of the enzyme activity detection kits (Keming Biotechnology Co., Ltd., Suzhou, China). The detection principles of enzyme activity of G6PD were that the enzymes can catalyze the reduction in NADP^+^ to generate NADPH. The enzyme activity of G6PD can be obtained by measuring the increase rate of NADPH at 340 nm. The activity unit of enzyme activity was defined as 1 nmol NADPH generated per mg of protein in the reaction system per minute.

When the cell extracts were prepared for NADPH content, adding 8 μL of perchloric acid with a concentration of 1.0 M was necessary before the fermentation liquid centrifugation, and then the cell extracts were prepared according to the above method. The NADPH content was detected based on the coenzyme II NADP (H) content kit (Keming Biotechnology Co., Ltd., Suzhou, China).

### 4.8. Quantitative Real-Time PCR (RT-qPCR) Assay

The *Schizochytrium* sp. H016 cells were collected at 72 h of fermentation, frozen with liquid nitrogen, and ground into fine powder. Total RNA was extracted using the Omega Fungi RAN extraction kit. The cDNA was synthesized by the Rever Tra Ace qPCR RT Master Mix with a gDNA Remover kit. The condition for RT-qPCR was according to the protocol of SYBR^®^ Green Realtime PCR Master Mix (Toyobo Co., Ltd., Osaka, Japan). The relative transcription levels of genes were calculated using the 2^-ΔΔCt^ method, and β-actin served as an internal control.

### 4.9. Statistical Analysis

The experimental samples were set up with three biological replicates, and the data were presented as mean ± SD. Data analysis was performed utilizing IBM SPSS Statistics 23 (IBM, New York, NY, USA), and *p* < 0.05, *p* < 0.01, and *p* < 0.001 levels indicate significant differences, which are denoted by *, **, and ***, respectively.

## 5. Conclusions

In conclusion, the results indicate that *G6PD* gene manipulation directly affects the supply of NADPH, a reducing force required for fatty acid synthesis, which in turn promotes lipid accumulation in *Schizochytrium* sp. H016. *G6PD* has been identified as an effective regulatory target for increasing DHA production in *Schizochytrium* sp. H016. This strategy will provide a strong technological basis for *Schizochytrium* sp. to increase the efficiency of producing high-value-added products and also providing useful information for other methods of oleaginous microbial biosynthesis of lipids and biofuels in significant quantities.

## Figures and Tables

**Table 1:** Comparison of the main fatty acid components of WT and OG6PD in fermentation medium. Data represent mean ± SD (*n* = 3).

Fatty Acid Composition	Fatty Acid Content (% of Total FA)		Fatty Acid Yield (g/L)	
	WT	OG6PD	WT	OG6PD
C14:0	1.65 ± 0.22	1.35 ± 0.01	0.21 ± 0.03	0.25 ± 0.01
C15:0	7.68 ± 1.46	3.32 ± 0.54	0.97 ± 0.18	0.61 ± 0.10
C16:0	36.33 ± 0.78	28.22 ± 0.16	4.57 ± 0.10	5.16 ± 0.03
C17:0	2.73 ± 0.43	1.20 ± 0.18	0.34 ± 0.05	0.22 ± 0.04
C18:0	1.39 ± 0.04	1.12 ± 0.08	0.17 ± 0.01	0.20 ± 0.01
EPA	0.50 ± 0.03	0.51 ± 0.07	0.06 ± 0.02	0.09 ± 0.01
DPA	8.79 ± 0.30	11.07 ± 0.01	1.10 ± 0.04	2.02 ± 0.02
DHA	36.64 ± 0.23	48.17 ± 1.10	4.60 ± 0.03	8.81 ± 0.20
SFA	49.77 ± 1.29	35.20 ± 0.64	6.25 ± 0.16	6.43 ± 0.12
PUFA	45.93 ± 0.49	59.75 ± 1.04	5.78 ± 0.07	10.92 ± 0.19

## Data Availability

Not applicable.

## Supplementary Materials

The following supporting information can be downloaded at: https://www.mdpi.com/article/10.3390/md21010017/s1, Table S1: Primers used in this study. Figure S1: The TLC of the lipid components of WT and OG6PD. Figure S2: GC and GC/MS pictures of the fatty acid methyl ester with indication of all fatty acid recognition with all standard references. Figure S3: GC enlargement of DHA area to visualize correctly the peak. Figure S4: GC/MS fragment of DHA.
