# Supplementary material for: Construction of Glucose-6-Phosphate Dehydrogenase Overexpression Strain of Schizochytrium sp. H016 to Improve Docosahexaenoic Acid Production
Source: Mar Drugs. 2022 Dec 26;21(1):17. doi: 10.3390/md21010017 (PMC9866257; doi:10.3390/md21010017)

Supplementary Materials

Table S1. Primers used in this study.

primers	sequences （5' ⟶ 3'）
EcoRI-AOX F	CGGAATTCCGTTTCGAATAATTAGT
XbaI-Myc R	GCTCTAGAACAAAAACTCATCTCAGAAGAG
18S U F	GCCAGTAGTCATATGCTCGTC
18S U R	GTCTTTGGATGTTAGATCTGCCAGAAATTCAACTAC
G6PDH F	CGGAATTCATGCTCAAAAGCTTTATCTC
G6PDH R	GCTCTAGATTACTTACTCTTGCCATGCTGC
AOX F	GTAGTTGAATTTCTGGCAGATCTAACATCCAAAGAC
CYC R	TGCCCTTCCGTCAATTCCGCAAATTAAAGCCTTCGA
18S D F	CGAAGGCTTTAATTTGCGGAATTGACGGAAGGGCA
18S D R	GAATCCAAACAGAAACATCC
β-actin F	ATCAAGGTCGTCGCTCCC
β-actin R	CACAGACTCGTCGTACTCCTG
PfaA F	ACCGTCTCGAGGCCAACATGGATAC
PfaA R	ATGAATCCACCACGTTTGCAGTAGATCTTA
PfaB F	CTGCCTGTGCCACCGCCCTTTA
PfaB R	TAGCACCGAGCAGAGTACCGTAAAT
PfaC F	CCATTGCCCCGCGTCTTAAC
PfaC R	ACGCGGTCAATCATGAGCATCTTA
FAS F	ACCAACGCAAGCTCGATGAGGATAT
FAS R	CTGCCCTCCTCTCCGAAGTCATCATAAC
PGAT F	ACACCGACGGTTATGTGCTT
PGAT R	GTGACATTTGTGCGTGTGCT
GPAT F	TCTGCTGCACATCGTGGTAG
GPAT R	GCAAGAAGAATGCGCCAGAG
HK F	CCGGACAGGTTCTCTTTGCT
HK R	TCCCTGTACCAAGCGTCAAC
PFK F	CGGATGCTGCTGCCATTAAC
PFK R	TAGGCAACCGGCACATTGAT
PK F	TAACACCCTCGCCAACATCC
PK R	CGTAGTCGGTGGTGATCTCG
PGD F	GACCGCACTGGATCTAAGGG
PGD R	GCAATGTAGCGAGCGTCAAG
PGLS F	TGGCTGCTGGCAAGAGTAAG
PGLS R	CCAGAGGTAGGACGAACACG
ACC F	TTCCGCCTCAACGCTATTGT
ACC R	GATACCGACAGAGCGACCAG

Figure S1: The TLC of the lipid components of WT and OG6PD.

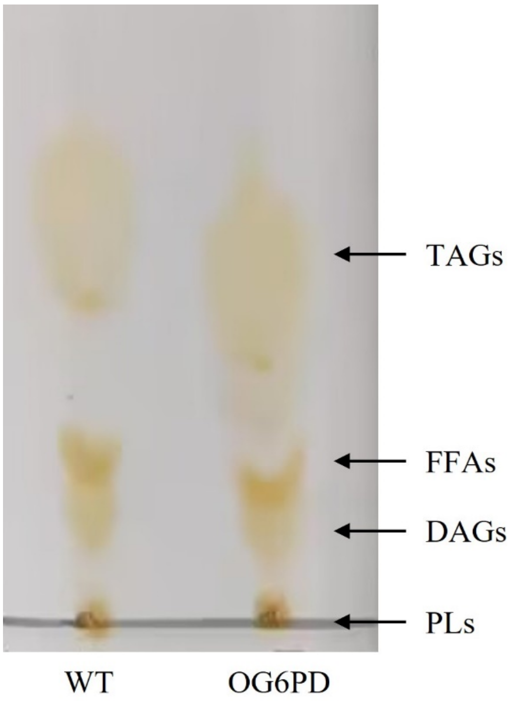

Figure S2: GC and GC/MS pictures of the fatty acid methyl ester with indication of all fatty acid recognition with all standard references.

The GC picture of all standard references:

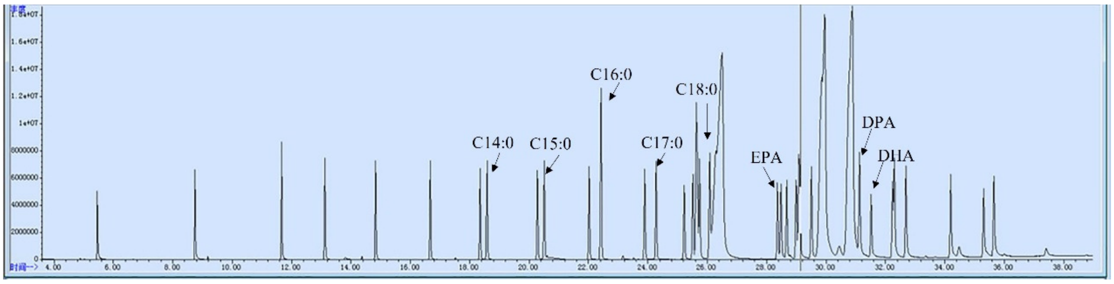

The GC/MS picture:

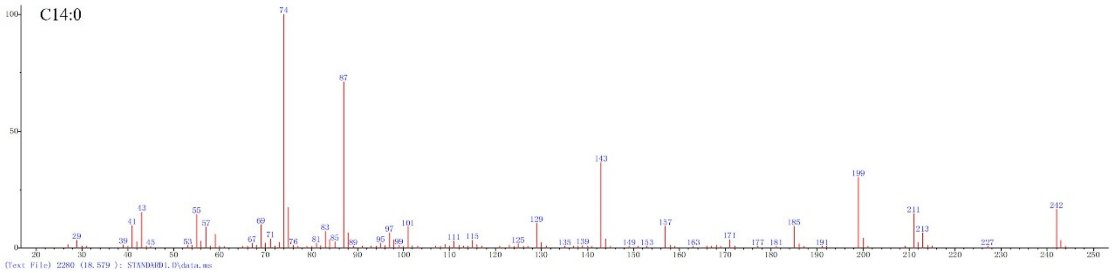

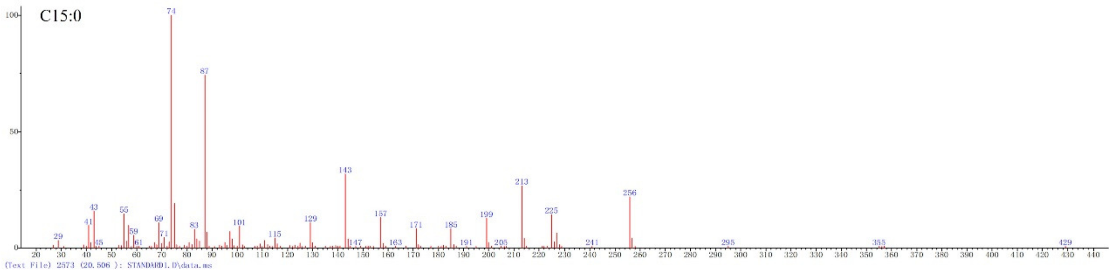

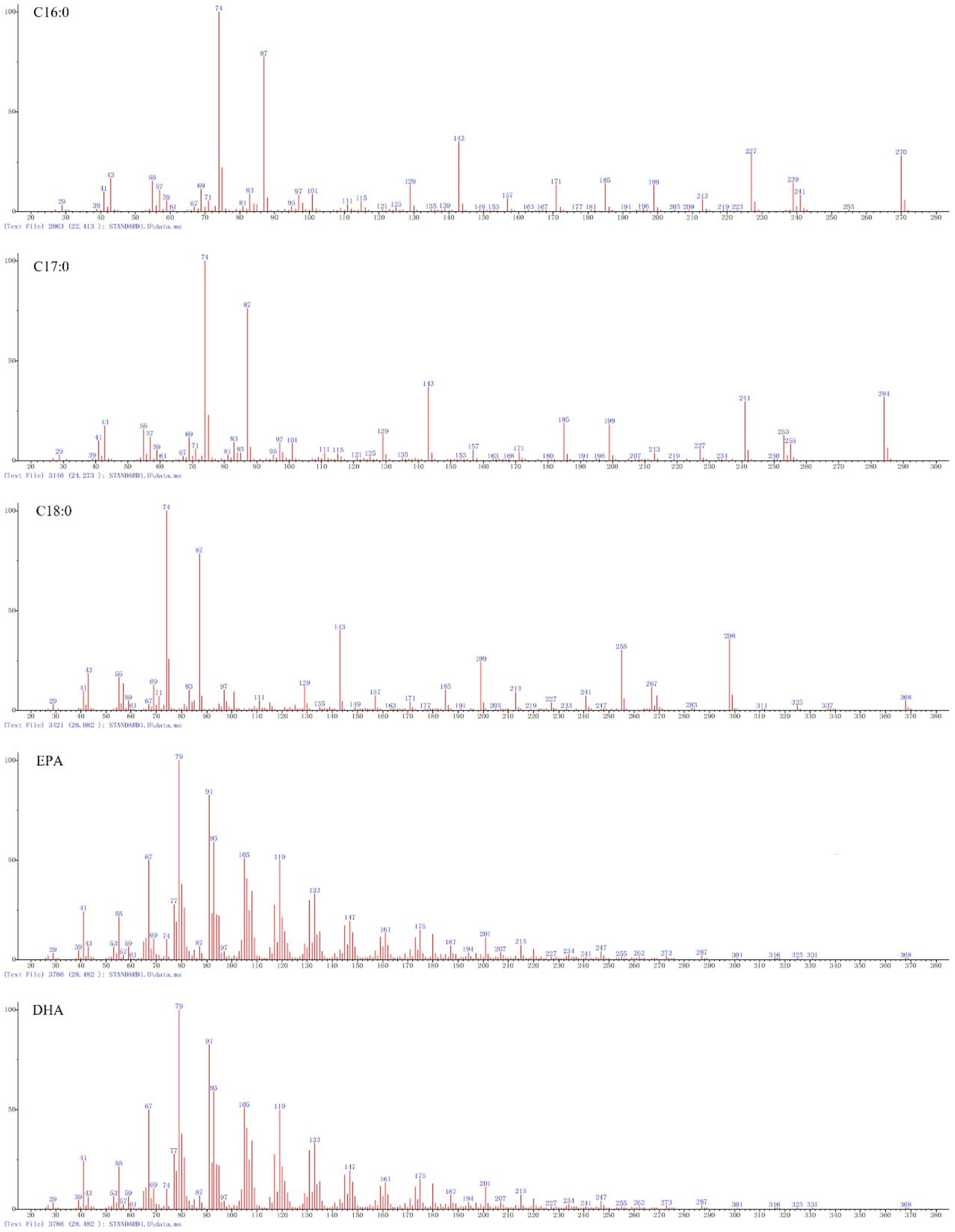

Figure S3: GC enlargement of DHA area to visualize correctly the peak.

WT:

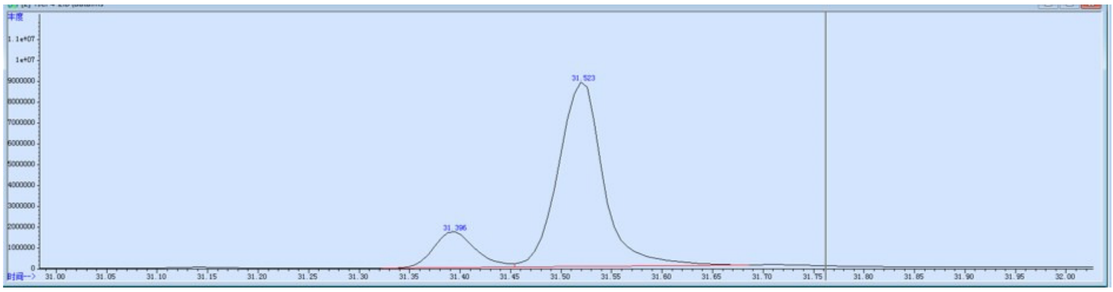

OG6PD:

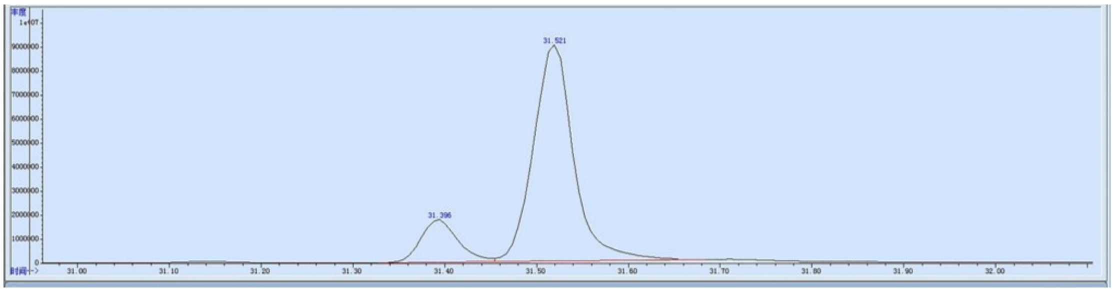

Figure S4: GC/MS fragment of DHA.

WT:

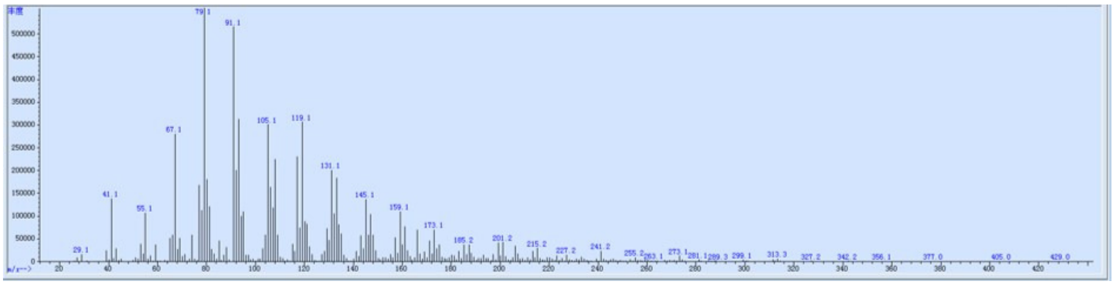

OG6PD:

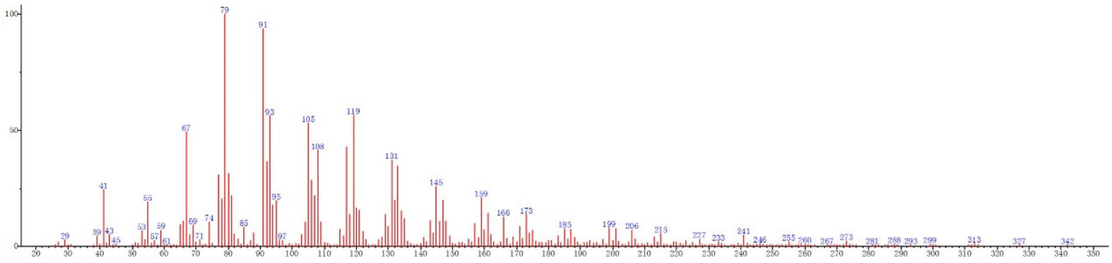

Supplement: Supplementary file 1 [file marinedrugs-21-00017-s001.zip › marinedrugs-2040227-supplementary.pdf]
